# Mechanisms of Pertussis Toxin Action: ADP-Ribosylation and Its Role in Pertussis Pathogenesis

**DOI:** 10.3390/toxins18030148

**Published:** 2026-03-18

**Authors:** Qing Tang, Ho Yung Chan, Yanxi Huang, Yung H. Wong

**Affiliations:** 1 Division of Life Science and the Biotechnology Research Institute, Hong Kong University of Science and Technology, Hong Kong, China; 2 Institute of Cancer Research, Shenzhen Bay Laboratory, Shenzhen 518132, China; 3 State Key Laboratory of Nervous System Disorders and the Daniel and Mayce Yu Molecular Neuroscience Center, Hong Kong University of Science and Technology, Hong Kong, China; 4 InnoHK Hong Kong Center for Neurodegenerative Diseases, Hong Kong, China

**Keywords:** pertussis toxin, ADP-ribosylation, Gi protein, cAMP, *Bordetella pertussis*

## Abstract

Pertussis toxin (PTx) is a major virulence factor of *Bordetella pertussis* and an AB5-type exotoxin that disrupts host signaling. Its enzymatic A subunit ADP-ribosylates the α-subunit of inhibitory G proteins (Gα_i_), preventing them from mediating receptor-induced inhibition of adenylyl cyclase (AC). This leads to unrestrained cAMP accumulation in host cells, a canonical mechanism underlying many pertussis disease manifestations. PTx works in concert with the bacterium’s adenylate cyclase toxin (ACT) to subvert immune defenses and establish infection. Interestingly, PTx exerts both cAMP-dependent and cAMP-independent effects. In addition to the well-known cAMP-mediated pathway, PTx’s B oligomer can engage host cell surface receptors to trigger signaling cascades independent of the A subunit’s catalytic activity. Such B oligomer-mediated pathways modulate cellular responses in the absence of ADP-ribosylation. This review provides a comprehensive analysis of PTx’s dual functionality, distinguishing its G_i_ protein-dependent elevation of cAMP from the noncanonical activities of the B oligomer. It also highlights how disruption of constitutive G_i_ signaling and the interplay between PTx and ACT shape host–pathogen interaction in pertussis pathogenesis.

## 1. Pertussis and Its Major Virulence Factors

Pertussis, commonly known as whooping cough, is an acute respiratory infection caused by the Gram-negative bacterium *Bordetella pertussis* (*B. pertussis*) [1]. Pertussis infection typically progresses through three clinical stages. The catarrhal stage is characterized by mild upper respiratory symptoms resembling those of a common cold. It is followed by the paroxysmal stage, during which patients experience spasmodic coughing fits often accompanied by inspiratory “whoop” (hence the name “whooping cough”), vomiting, or transient cyanosis. Finally, in the convalescent stage, the frequency and severity of coughing gradually diminish, although symptoms may persist for several weeks.

*B. pertussis* is a strictly human pathogen that has evolved a specialized arsenal of virulence factors to colonize the respiratory tract and cause disease [2]. It preferentially attaches to the ciliated epithelium of the upper airway, where the bacteria multiply on the surface of host cells, evade mucociliary clearance and immune defenses, and ultimately damage the respiratory epithelium.

**Figure 1:**
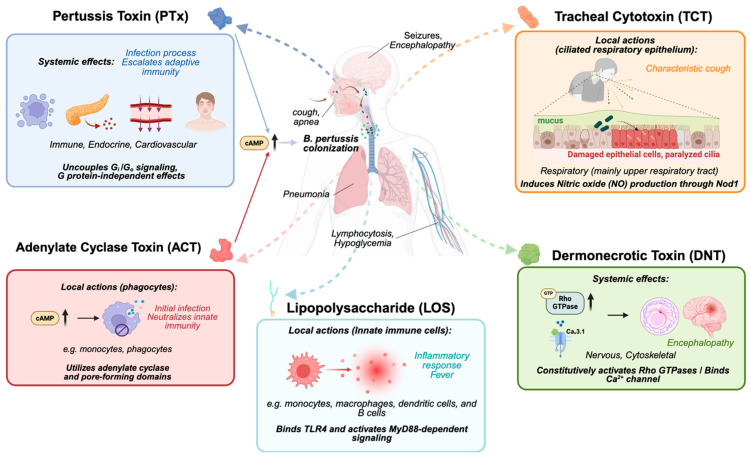
An overview of the toxins produced by *B. pertussis* and their target organs/systems. PTx acts primarily as a systemic effector, uncoupling G_i/o_ protein signaling and modulating immune, endocrine, and cardiovascular responses, thereby modifying adaptive immunity and contributing to lymphocytosis and hypoglycemia. ACT functions locally on phagocytes, where calmodulin-activated cAMP bursts disrupt innate immune functions such as phagocytosis and pore formation. TCT targets the ciliated respiratory epithelium, causing epithelial damage, impaired mucociliary clearance, and the characteristic paroxysmal cough. DNT constitutively activates Rho GTPases and modulates Ca^2+^ channels, inducing cytoskeletal alterations and contributing to neurological symptoms. LOS signals through TLR4–MyD88 pathways and triggers strong local inflammatory responses in innate immune cells. Together, these toxins shape *B. pertussis* colonization, immune evasion, tissue damage, and systemic disease manifestations.

Pertussis toxin (PTx) is a major virulence factor secreted by *B. pertussis* and is a key contributor to the pathogenesis of whooping cough. The diverse pathological effects of pertussis are primarily attributed to PTx, a view consolidated from several biological activities that were historically described and investigated as separate phenomena: histamine-sensitizing factor (HSF) [3], lymphocytosis-promoting factor (LPF) [4], and islet-activating protein (IAP) [5]. The definitive link between these activities was established by Munoz et al. [6], who examined a highly pure, crystalline protein that had been previously isolated from the *B. pertussis* Tohama I strain [7]. Their research demonstrated conclusively that this single protein, which they named pertussigen (now known as PTx), was responsible for all three effects, establishing that nanogram amounts could induce the characteristic physiological changes in mice (e.g., leukocytosis and increased insulin secretion), while microgram doses were lethal [6]. Early physiological studies by Katada and Ui further showed that pretreatment of rats with *B. pertussis* vaccine abolished α_2_-adrenergic receptor-mediated inhibition of insulin secretion [8]. This key finding revealed that PTx selectively disrupts G_i_-coupled receptor function in vivo, a concept later summarized by Katada [9]. While PTx is central to many systemic symptoms, it acts in concert with other toxins released by *B. pertussis*, such as adenylate cyclase toxin (ACT), tracheal cytotoxin (TCT), dermonecrotic toxin (DNT), and lipooligosaccharide (LOS), that collectively orchestrate the disease’s complex pathology [10,11,12,13,14,15] (Figure 1).

ACT, also known as adenylate cyclase toxin-hemolysin (CyaA), increases intracellular cAMP levels in host cells, particularly targeting phagocytes through CD11b/CD18 heterodimer [11,12]. It has two main functions: an adenylate cyclase domain that binds to host calmodulin to generate excessive cAMP and a domain that creates cation-selective pores in host cell membranes [16]. This dual action impairs the ability of immune cells to destroy bacteria by inhibiting monocyte-to-macrophage transition, and high amount of ACT can induce cell death [16,17]. While ACT exhibits high-affinity binding to the CD11b/CD18 integrin on phagocytes, its membrane permeability also allows for receptor-independent entry into a wider variety of host cells [18]. This dual-targeting capability complements the systemic effects of PTx, creating a potent “one-two punch” against the host immune response [11]. Both toxins function by dramatically elevating intracellular cAMP to suppress key innate immune functions like phagocytosis and inflammatory cytokine production [19,20]. This synergy is reflected in their evolution; *B. pertussis* produces less ACT than its relative *B. bronchiseptica*, likely because the potent, systemic immunosuppression by PTx lessens the need for high quantities of ACT to disable phagocytes [21].

Unlike the other protein toxins, TCT is a fragment of the bacterium’s peptidoglycan cell wall that is released during cell growth [13,22]. It is specifically toxic to the ciliated epithelial cells that line the respiratory tract, causing these cells to stop beating and can lead to their extrusion from the lining, which impairs the clearance of mucus and debris from the lungs and contributes to the characteristic cough of pertussis [23]. DNT is a potent intracellular toxin that permanently activates the host cell’s Rho GTPases, leading to severe disruption of the cellular cytoskeleton [14,24]. More importantly, DNT has been identified as a neurotropic virulence factor that specifically targets nerve cells by binding to the CaV3.1 calcium channel receptor, which might be the cause of pertussis encephalopathy, a devastating neurological complication of whooping cough [25]. As a Gram-negative bacterium, *B. pertussis* has LOS in its outer membrane, which can be released to act as an endotoxin [15,26]. The unique structure of *B. pertussis* LOS contributes to the inflammatory response and fever associated with the infection. In addition to these toxins, *B. pertussis* also produces several other virulence factors, such as filamentous hemagglutinin, fimbriae, and pertactin, which act as adhesins to help the bacteria attach to the cells of the respiratory tract (Table 1).

## 2. The Canonical Actions of PTx: Gα_i_- and cAMP-Dependent Mechanisms

### 2.1. Structural Organization of PTx

**Figure 2:**
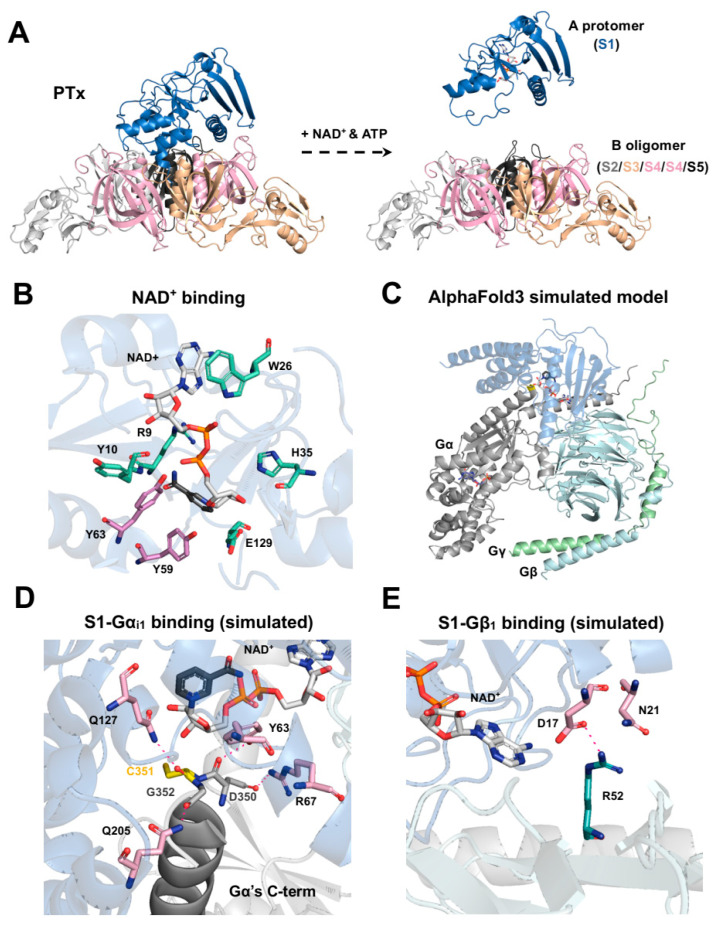
Structural features of PTx and its interaction with NAD^+^, Gα_i1_, and Gβ_1_. (**A**) The full PTx structure (PDB code: 1PRT) is shown, including the S1 catalytic subunit (blue) and the B-oligomer, composed of S2 (gray), S3 (pale orange), S4 (pink), and S5 (black) in a 1:1:2:1 ratio. In the cytosol, ATP facilitates dissociation of the S1 subunit (PDB code: 7SKY) from the B-oligomer, coupled with the cleavage of the disulfide bond between C41 and C201, enabling NAD^+^ and Gα to access the catalytic domain. (**B**) Key residues involved in ADP-ribosyltransferase activity (R9, Y10, W26, H35, and E129) are shown in green. Residues critical for Gα binding (Y59 and Y63; [58]) are highlighted in pink. (**C**) Docking simulations using AlphaFold3 incorporated S1, Gα_i1_, Gβ_1_, Gγ_2_, NAD^+^, and GDP. Consistent structural models were observed only when Gβ_1_ and Gγ_2_ were included, consistent with prior studies [29,47,59]. (**D**) Key residues forming hydrogen bonds between S1 and Gα_i1_ in the selected docking model (**C**) are shown. (**E**) Key residues forming hydrogen bonds between D17 and N21 residues of S1 and R52 of Gβ_1_ are shown.

Structurally, PTx is a heterohexameric complex with a molecular weight of approximately 105 kDa, composed of five distinct subunits: S1 (26 kDa), S2 (22 kDa), S3 (22 kDa), two copies of S4 (12 kDa), and S5 (10 kDa) [27] (Figure 2A). The holotoxin conforms to the classical AB-type toxin model, in which the catalytically active A protomer (S1) possesses ADP-ribosyltransferase activity [46], while the B oligomer, formed by the S2, S3, S4, and S5 subunits, mediates high-affinity binding to glycoprotein receptors on the host cell surface [47].

Upon binding to glycoprotein receptors, the holotoxin is internalized via endocytosis and retrogradely transported through the endosomal–Golgi network, ultimately reaching the endoplasmic reticulum (ER) [48]. Within the ER, ATP binding to the B oligomer triggers the dissociation of the S1 subunit from the complex [49]. The liberated S1 subunit is thermolabile at physiological temperature, rapidly adopting a partially disordered conformation that prevents its reassociation with the B oligomer. This unfolded state enables S1 to hijack the ER-associated degradation pathway, facilitating its retro-translocation across the ER membrane into the cytosol [50]. In the cytoplasm, glutathione reduces the conserved C41–C201 disulfide bond within S1, exposing the catalytic core and thereby activating the enzyme [28].

This modular architecture reflects a clear division of labor between the binding (B) and catalytic (A) components: the B oligomer ensures precise cellular targeting and delivery, whereas the S1 subunit, once in the cytosol, enzymatically modifies host G proteins. Such structural organization underlies the efficient intoxication of host cells and forms the molecular basis of the canonical ADP-ribosylation mechanism of PTx.

### 2.2. Enzymatic Mechanism of Gα_i_ ADP-Ribosylation

In host cells, heterotrimeric G proteins (Gαβγ) serve as central mediators of GPCR signaling [51]. The Gα subunit binds and hydrolyzes guanine nucleotides, while the Gβγ complex acts as a membrane anchor that regulates multiple downstream effectors. In the resting state, Gα remains bound to GDP and associates with Gβγ to form an inactive trimeric complex. Upon receptor activation by an agonist, the GPCR catalyzes the exchange of GDP for GTP on Gα, promoting its dissociation from Gβγ [52]. The released Gα–GTP and free Gβγ subunits then modulate a variety of effectors [51]. Among G protein subfamilies, the G_i/o_ family plays an inhibitory regulatory role: activated Gα_i_ suppresses AC catalytic activity to reduce intracellular cAMP levels.

The primary molecular targets of S1 are the α-subunits of inhibitory heterotrimeric G proteins (G_i_, G_o_) and the visual transduction protein transducin (G_t_) [5,30,53]. PTx catalyzes the transfer of ADP-ribose from NAD^+^ to a conserved cysteine residue, typically four residues from the C-terminus, of these α subunits [30,53]. Notably, PTx prefers to catalyze trimeric G_i_ proteins that consist of Gαβγ subunits, rather than the Gα monomer alone [54,55,56]. This covalent modification on the cysteine residue of Gα_i/o/t_ subunits prevents their coupling to receptors, thereby disabling receptor-mediated GDP/GTP exchange. In cell-free systems, PTx activity requires NAD^+^ and ATP, with nicotinamide as a reaction byproduct [31]. Radiolabeled NAD^+^ tracing confirmed that the ADP-ribose moiety, but not nicotinamide, is incorporated into a 41 kDa membrane protein, later identified as Gα_i_, with the modification correlating with enhanced AC activity [32,57].

Mutagenesis and crystallographic studies have revealed how a network of key residues in the S1 subunit exert its enzymatic activity (Table 2). Its catalytic core has a conserved structural feature involving Y8 and E129 [28], which can also be found in cholera toxin (Y6 and E112) [60], exotoxin A (Y439 and E553) [61], and diphtheria toxin (Y20 and E148) [62]. Within this pocket, E129 acts as the key essential glutamate to destabilize NAD^+^ [63,64,65,66], while H35 primes the target cysteine on Gα for nucleophilic attack [67,68]. The integrity of this active site is further supported by Q127 [58,69] and S52 [67], which help maintain its precise geometry. The NAD^+^ is bound by interactions with R9, Y10, W26, H35, L36, Q42, S52, T53, Y63, and Y130 [63,65,70,71,72,73,74] (Figure 2B). For recognizing the Gα_i_ protein, the subunit relies on hydrophobic contacts from Y59 and Y63 [58], along with the broader 195–204 region [75]. On the substrate side, a conserved cysteine four amino acids away from the carboxyl terminus of the Gα_i_ subunit is essential for modification, substitutions or positional shifts weaken or even abolish ADP-ribosylation [58]. Structural modeling using AlphaFold3 [76] (Figure 2C) (using each of PTx S1, Gα_i1_, Gβ_1_, Gγ_2_, NAD, and GDP for docking) further suggests that residues D350, C351, and G352 in the C-terminal helix of Gα_i1_ could form potential hydrogen bonds with Y63, R67, Q127, and Q205 of S1, orienting the acceptor cysteine into the catalytic pocket (Figure 2D). Through ADP-ribosylation, PTx (S1) irreversibly prevents G_i/o_-mediated signaling, potentially increasing the intracellular cAMP level, which is a key biochemical event underlying many downstream effects in pertussis pathogenesis. Collectively, these structural and biochemical insights delineate how PTx catalyzes a precise and irreversible ADP-ribosylation reaction that selectively targets G_i_ proteins, abolishes inhibitory signaling, and drives persistent cAMP elevation—defining the canonical mechanism of PTx action.

### 2.3. cAMP-Mediated Effects and Pathophysiological Outcomes

**Figure 3:**
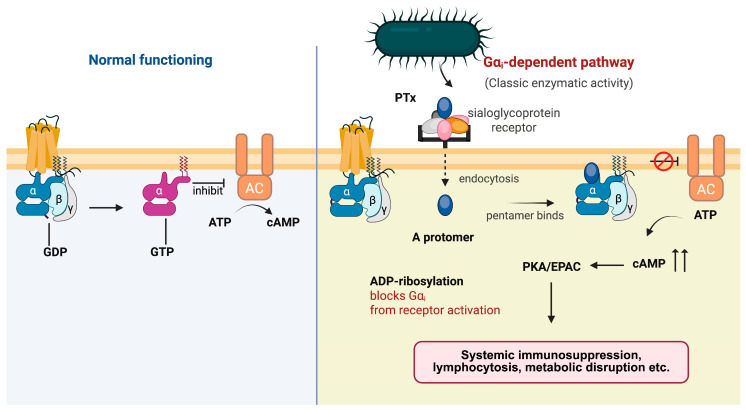
Canonical cAMP signaling cascade triggered by PTx. Under normal conditions, Gα_i_ couples to its receptor and inhibits AC, thereby restraining intracellular cAMP production. Upon exposure to PTx, the A protomer enters host cells and catalyzes ADP-ribosylation of Gα_i_, preventing receptor-mediated activation of the G protein. Loss of functional Gα_i_ releases tonic inhibition of AC and results in excessive cAMP accumulation. Elevated cAMP activates downstream PKA and EPAC pathways, leading to systemic immunosuppression, lymphocytosis, and metabolic disruption.

By inactivating G_i_ and thereby releasing the inhibitory brake on AC, PTx drives a persistent rise in intracellular cAMP (Figure 3). This chronic cAMP elevation engages two major cAMP-responsive signaling branches, protein kinase A (PKA) [79] and the exchange proteins directly activated by cAMP (EPAC) [80], which together reprogram multiple immune and metabolic pathways.

In immune cells, elevated cAMP functions as a potent immunosuppressive signal. Increased cAMP suppresses leukocyte integrin activation as well as adhesion and migratory capacity through PKA, whereas EPAC activation inhibits the production of pro-inflammatory cytokines and chemokines [20]. In the endocrine system, elevated cAMP stimulates insulin secretion from pancreatic β-cells, a mechanism that explains the hypoglycemia frequently observed during severe pertussis infection [81]. Similarly, in vascular and neuronal tissues, the loss of G_i_-mediated inhibitory tone heightens responsiveness to vasoactive amines such as histamine, leading to exaggerated vasodilation and histamine hypersensitivity—classical bioassay readouts of PTx activity. Gα_i1_ and Gα_i3_ constitute the key molecular targets mediating this response, as genetic ablation of either subunit phenocopies PTx exposure and renders mice spontaneously hypersensitive to vasoactive amine sensitization (VAAS) [82].

## 3. Synergistic Interplay and Dominant Dynamics Between PTx and ACT

The canonical enzymatic mechanism of PTx establishes the fundamental basis for G_i_ inactivation and sustained elevation of intracellular cAMP. However, in infected hosts, this process rarely acts in isolation. *B. pertussis* secretes multiple toxins that converge upon the same second-messenger network, among which ACT plays a particularly prominent role. Together, PTx and ACT constitute a synergistic dual-toxin strategy that amplifies and maintains intracellular cAMP accumulation across distinct immune cell populations. This section describes how these two toxins cooperatively disrupt cAMP homeostasis, the resulting immunopathological consequences, and the key determinants that modulate their interplay.

### 3.1. Toxin Dominance and Disease Subtypes: From PTx-Dominant to ACT-Dominant Infections

Currently, no standardized method exists to directly quantify the relative secretion levels of PTx and ACT. Because the expression of both toxins is shaped by the BvgAS regulatory state, strain genotype, host environment, and stage of infection, their relative dominance can only be inferred indirectly through multiple complementary readouts: (1) transcriptional and protein-level measurements, such as comparative expression of ptxA/B/E and cyaA [83,84]; (2) functional assays, including PTx-mediated Gα_i_ ADP-ribosylation activity and the measurement of intracellular cAMP peaks induced by PTx or ACT [85,86]; (3) host response signatures, such as leukocyte apoptosis, phagocytic suppression, and characteristic immunopathological features observed in experimental or clinical models.

In the context of cell death, ACT exhibits clear predominance. ACT induces rapid apoptosis in macrophages, neutrophils, and dendritic cells [87]. In the mouse lung, strains producing ACT trigger pronounced macrophage and neutrophil apoptosis, whereas ACT-deficient mutants fail to do so [88]. In contrast, PTx does not directly induce apoptosis in phagocytes; its major effects in lymphoid and myeloid cells manifest as disruptions in migration and homing rather than acute loss of survival signals. The extremely high intracellular cAMP levels generated by ACT profoundly impair macrophage and neutrophil phagocytic capacity [89] and suppress the oxidative burst response [90]. Correspondingly, ACT-deficient strains restore normal phagocytic function in the same experimental models [91]. In tissues affected by ACT, one observes a characteristic pattern of acute pulmonary inflammation, epithelial barrier damage, exudative injury, and paradoxically poor inflammatory cell infiltration, a phenotype marked by severe local tissue injury but ineffective immune clearance.

At the level of immunopathology, PTx disrupts basal inhibitory signaling across multiple tissues through irreversible ADP-ribosylation of Gα_i/o_, thereby provoking a broad spectrum of immune and physiological dysregulation. The most characteristic in vivo readout is the marked peripheral lymphocytosis, whose magnitude strongly correlates with disease severity and strictly depends on the enzymatic activity of PTx [92]. Comparative studies with *B. parapertussis*, a closely related species lacking PTx, similarly demonstrate a greatly diminished or absent lymphocytosis response [93]. Another PTx-specific immunopathological hallmark is VAAS, a phenomenon that underlies the classical bioassay used to assess the potency of pertussis vaccines and purified toxin preparations [94].

Taken together, leukocyte apoptosis, phagocytic suppression, and distinct immunopathological signatures provide reliable markers to discriminate PTx-dominant from ACT-dominant effects. PTx primarily drives systemic immune dysregulation and defects in cell trafficking, whereas ACT triggers acute cellular injury and profound suppression of phagocytic function.

### 3.2. Determinants of Dominance: Temporal Window and Dose Ratio

The relative dominance of PTx and ACT is not fixed but shifts dynamically over the course of infection, shaped by temporal windows and dosage. ACT exerts its effects on a remarkably rapid timescale, typically within minutes to hours after contacting host cells. As a single-component RTX toxin secreted directly through the type I secretion system, ACT rapidly binds to target cell membranes and, upon calmodulin activation, immediately triggers a sharp, localized burst of cAMP. This rapid signaling spike directly suppresses macrophage and neutrophil phagocytosis, oxidative burst activity, and migratory capacity, producing a characteristic frontline immune paralysis. In *B. pertussis* cultures, ACT secretion is detectable at 60 min. and plateaus by 6 h [95]. Experimentally, elevated cAMP can be detected within seconds post infection [96], oxidative burst is inhibited within 5 min [97], phagocytic suppression becomes evident within 1 h [98], and apoptosis appears by 4 h [99].

**Figure 4:**
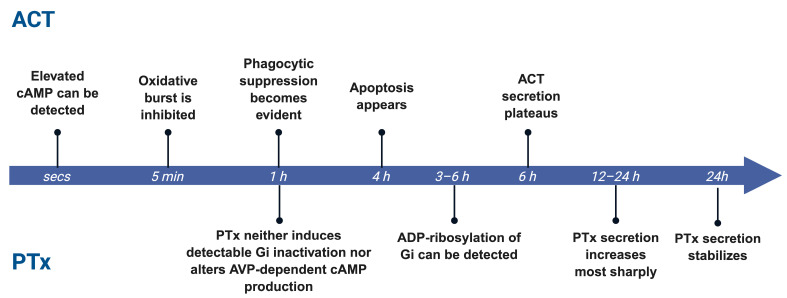
Temporal separation of ACT and PTx activity. ACT acts rapidly within minutes to hours, whereas PTx shows delayed activity requiring prolonged exposure.

In contrast, the bioactivation of PTx is markedly delayed. Studies in cells and tissues show that PTx requires several hours of uptake and intracellular trafficking before it can ADP-ribosylate G_i_ proteins and induce stable signaling blockade. For example, in renal tubule and thyroid tissue slices, and specifically in isolated medullary collecting tubules and medullary thick ascending limbs, PTx requires at least 3–6 h of preincubation to ADP-ribosylate Gα_i_; brief 1 h exposure neither induces detectable G_i_ inactivation nor alters arginine vasopressin-dependent cAMP production [100]. In *B. pertussis* culture, PTx secretion increases most sharply between 12 and 24 h and then stabilizes [101] (Figure 4). This temporal division of labor ensures that ACT acts first to disable local immune defenses, while PTx subsequently reinforces systemic immune dysregulation through long-range signaling mechanisms, thereby sustaining persistent infection.

The effective environmental concentration of ACT typically falls within the tens to hundreds of ng/mL range; 60–80 ng/mL in in vitro cultures, and ~12–20 ng/mL in nasopharyngeal aspirates from infected infants [95]. In contrast, PTx exhibits strong biological activity at substantially lower concentrations, often in the ~1–10 ng/mL range, as demonstrated by its disruption of Gα_i_ signaling [102]. PTx also displays much greater diffusibility and systemic “spillover” capacity: even a small subpopulation of PTx-producing bacteria can secrete the toxin at levels sufficient to act on distant host cells and tissues, amplifying its overall impact. Experimental observations show that, in mixed infections, a minority of PTx-secreting strains can enhance the colonization capacity of neighboring non-producing strains; wild-type PTx^+^ *B. pertussis* can secrete diffusible toxin in vivo that compensates for the colonization defect of PTx-deficient mutants [103]. In contrast, the effects of the cytolytic ACT are highly concentration dependent and remain confined to the microenvironment where the toxin directly contacts host cells. In mixed infections, PTx^+^ bacteria can “cover” or complement PTx^−^ strains, whereas ACT activity remains restricted to dense, localized lesions [11].

### 3.3. Pathological and Therapeutic Implications

In pathophysiological states dominated by PTx, systemic manifestations are the most pronounced. In infants, severe lymphocytosis and pulmonary hypertension are strongly associated with PTx activity [104,105]. Moreover, passive immunization targeting PTx reduces disease severity and improves systemic outcomes [106].

In addition to supportive care, clinical management may include (1) early passive immunization or administration of anti-PTx antibodies to block toxin entry into host cells [107]; (2) exchange transfusion or leukapheresis for life-threatening hyperleukocytosis, enabling rapid reduction in pulmonary arterial pressure [108]; and (3) antihistamines mitigate PTx-mediated vascular hypersensitivity and hypotension [109]. Together, these interventions target the PTx-induced inactivation of G_i_ proteins and the resulting systemic imbalances in signal transduction.

In ACT-dominant infections, localized immune dysfunction is a defining feature. For example, patients infected with *B. parapertussis* typically exhibit mild symptoms and rarely develop marked leukocytosis [93]. Studies have shown that anti-ACT antibodies can restore neutrophil phagocytosis and bactericidal activity [110]. Moreover, several TLR agonists and mucosal adjuvants, such as TLR2/TLR4 agonists, can elicit strong Th1/Th17 responses and mucosal IgA production in experimental models, thereby reducing *B. pertussis* colonization [111,112]; conceptually, these responses could counteract ACT-mediated local immunosuppression, although most evidence remains indirect.

## 4. Suppression of Basal G_i_ Activity by PTx

In the classical model, GPCRs are thought to engage G_i_ proteins only upon ligand binding, thereby inhibiting AC and reducing intracellular cAMP levels. However, accumulating evidence indicates that many cell types maintain a measurable level of G_i_-mediated inhibitory tone even in the absence of exogenous agonists. This so-called constitutive G_i_ signaling, or basal G_i_ activity, is now recognized as an intrinsic “tension” essential for maintaining signaling homeostasis and metabolic equilibrium [113].

### 4.1. Experimental Evidence for Basal GPCR Signaling and PTx Sensitivity

A key prediction of constitutive G_i_ signaling is that, in the presence of a pre-existing inhibitory tone, PTx should elevate basal cAMP levels, resulting in a detectable increase in cAMP even in the absence of GPCR agonists. We validated this concept using a cAMP biosensor assay in HEK293 cells transiently expressing the cAMP-Glosensor-22F reporter (a cAMP-sensitive bioluminescent sensor). Cells were transfected with either an empty vector or plasmids encoding representative GPCRs with distinct coupling preferences, including the G_s_-coupled β_2_-adrenergic (β_2_-AR) and D_1_ receptors (D_1_R), G_i_-coupled dopamine D_2_ receptor (D_2_R), and melatonin MT_1_ and MT_2_ receptors (MT_1_R and MT_2_R; known to exhibit constitutive G_i_ activity) [114].

**Figure 5:**
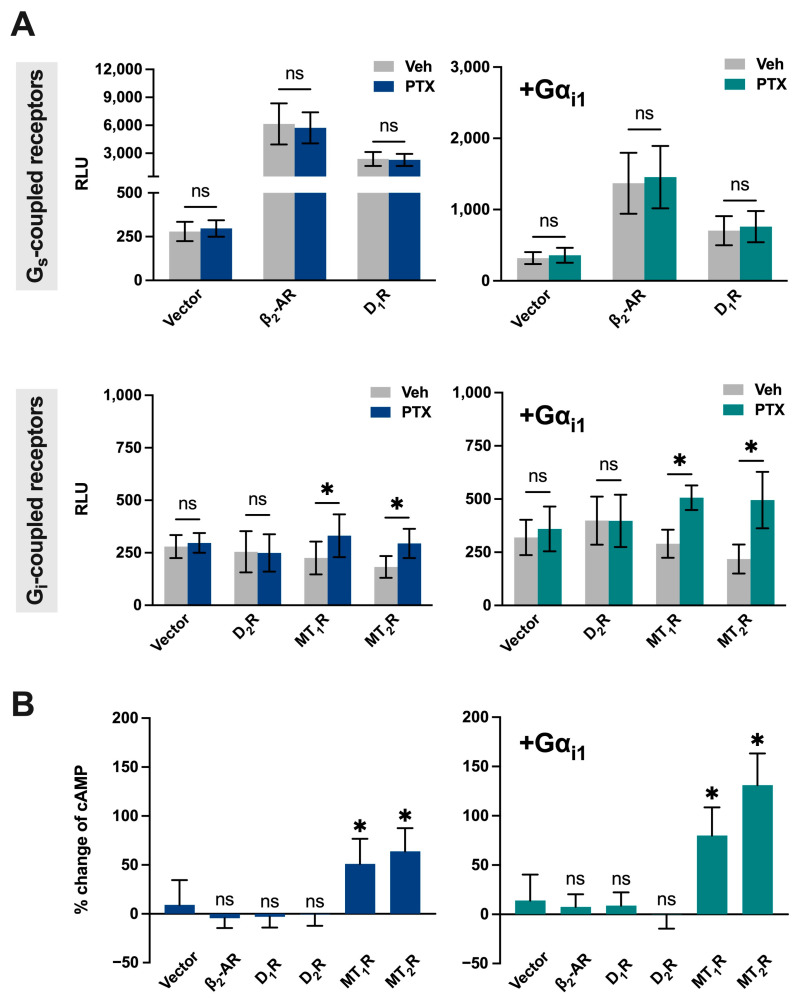
PTx-induced cAMP elevation depends on receptor constitutive activity and G_i_ abundance. (**A**) Left: Baseline cAMP levels (luminescence in RLU) in HEK293 cells transiently transfected with cAMP-Glosensor-22F (22F), one of the indicated GPCRs, and vector (pcDNA3.1) with or without PTx pretreatment. Each construct was used at 0.4 µg per well (total 1.2 µg DNA/well in a 6-well plate). Top: β_2_-AR or D_1_R. Bottom: D_2_R, MT_1_R, or MT_2_R. Right: Baseline cAMP levels measured same as the left, except that cells were co-transfected with Gα_i1_. (**B**) Percent increase in cAMP upon PTX treatment. Left: Cells with endogenous G_i_ background. Right: Cells overexpressing Gα_i1_. Data are extracted from panel A; *n* = 3; data are shown as mean ± SD. An asterisk (*) indicates a significant difference (*p* ≤ 0.05), and ‘ns’ indicates no significance (*p* > 0.05).

In cells transfected with MT_1_R or MT_2_R, we observed that PTx treatment led to a marked increase in basal cAMP levels (Figure 5A; bottom left). This effect is likely due to the constitutive activity of MT_1_R and MT_2_R on G_i_ activity; disruption of constitutive G_i_ signaling by PTx relieved the inhibitory constraint on AC, resulting in elevated cAMP. Furthermore, in cells with increased availability of Gα_i1_ by co-transfection, the PTx-induced increase in cAMP became more pronounced (Figure 5A; bottom right). This suggests that the magnitude of constitutive inhibition of AC is dependent on the level of G_i_ available to MT_1_R and MT_2_R. Interestingly, another G_i_-coupled receptor, D_2_R, did not exhibit any increase in cAMP upon PTx treatment, suggesting that not all GPCRs possess constitutive activity (Figure 5A, bottom).

Constitutive activity was also observed with the G_s_-coupled β_2_-AR and D_1_R since their expression significantly increased basal cAMP levels, which were unaffected by PTx treatment (Figure 5A, top left). The lack of effect by PTx reflected that these two receptors are predominantly coupled to G_s_. Nevertheless, the constitutive activities of β_2_-AR and D_1_R were attenuated upon increased expression of Gα_i1_ (Figure 5A, top right). Overall, these results indicate that PTx can directly cause significant changes in cAMP levels, particularly in cells expressing constitutively active G_i_-coupled receptors and/or with a high G_i_ availability back-ground (Figure 5B). Our findings align with a growing body of research that suggests that certain GPCRs exhibit constitutive activity, especially among orphan, melatonin, and chemokine receptors [114,115,116].

### 4.2. Mechanistic Models of PTx-Induced Disruption of Basal G_i_ Control

**Figure 6:**
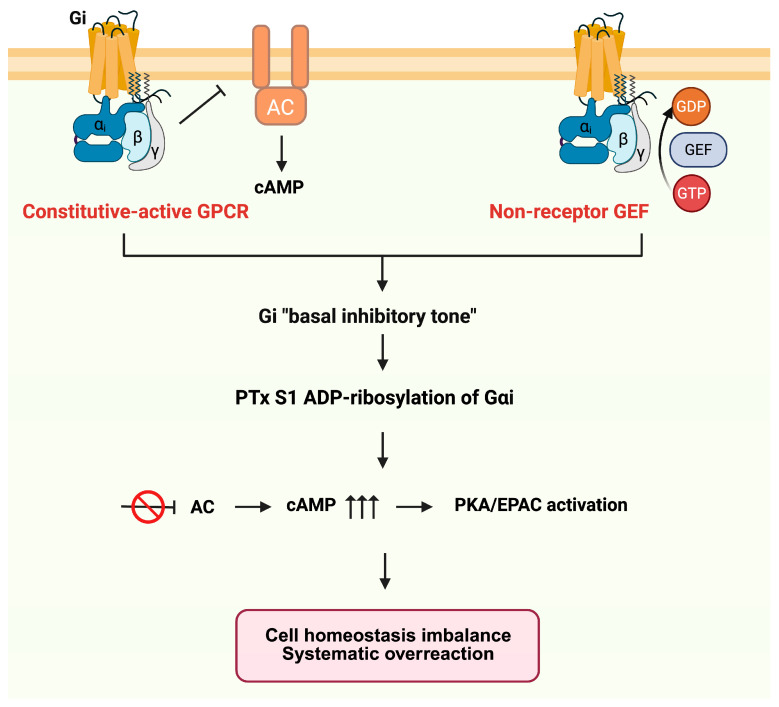
Mechanistic models of PTx disruption of basal G_i_ inhibitory tone. Basal G_i_ activity can arise from constitutively active or autocrine-stimulated G_i_-coupled GPCRs (left), or from receptor-independent mechanisms such as non-receptor GEFs or lipid-driven G_i_ activation (right). PTx-mediated ADP-ribosylation of Gα_i_ eliminates this inhibitory tone, leading to AC disinhibition, elevated cAMP, and downstream PKA/EPAC overactivation. Arrows indicate signaling flow, and the inhibition symbol indicates suppression of AC activity.

To explain how PTx disrupts this basal inhibitory tone, we propose two complementary mechanistic models (Figure 6). Firstly, some G_i_-coupled GPCRs can exert a persistent inhibitory input to AC even in the absence of exogenous agonists, as demonstrated for the melatonin receptor. This G_i_-dependent basal constraint on AC activity is thus sensitive to PTx treatment [117]. For example, the melatonin MT_1_R displays ligand-independent (constitutive) activity toward G_i_. Orphan GPCRs, such as GPR20 also exhibit robust G_i_/cAMP-mediated constitutive activity [118]. Importantly, disruption of basal G_i_ signaling by PTx may not only relieve tonic inhibition of AC but also unmask latent stimulatory signaling pathways. Recent work has demonstrated that certain G_i_-coupled GPCRs can exhibit context-dependent coupling to G_s_, wherein ADP-ribosylation of G_i_ reveals a PTx-insensitive stimulatory component. Notably, the melatonin MT_1_R —while maintaining constitutive G_i_-mediated inhibition of AC—was shown to potentiate forskolin-stimulated cAMP accumulation and, under conditions of elevated G_s_ availability, to directly stimulate cAMP production in a PTx-insensitive manner, highlighting a dual coupling behavior that amplifies cAMP responses following G_i_ inactivation [119].

In addition, receptors activated by autocrine ligands can maintain continuous G_i_ signaling. Many cell types employ autocrine loops in which G_i_-coupled receptor agonists, such as adenosine [120], prostaglandins [121], or sphingosine-1-phosphate (S1P) [122], are produced locally and can tonically activate G_i_ pathways. Notably, pro-inflammatory cytokines such as TNF-α can further enhance this basal G_i_ activity by upregulating S1P production [123], thereby sustaining the activation state of G_i_-coupled receptors. Thus, in cells lacking constitutive or autocrine G_i_ input, PTx has only minimal effects on cAMP levels. If no GPCR engages G_i_ under resting conditions, there is no persistent inhibitory tone to remove, underscoring that the impact of PTx depends on the presence of such receptor-driven basal signaling.

Secondly, basal G_i_ activity can also arise independently of ligand-bound receptors through intrinsic regulatory mechanisms of the G protein itself. G_i_ proteins can be activated by non-receptor guanine nucleotide exchange factors (GEFs), such as the non-receptor GEF Ric-8A [124]. The membrane lipid environment can markedly enhance pre-coupling and binding stability between GPCRs and G_i_ [125]. Moreover, recent work has demonstrated that not all forms of constitutive G_i_ activity are functionally equivalent. Using systematic chimeric and mutational analyses, Chung et al. showed that receptor-activated Gα_i_ adopts a distinct and more stable inhibitory conformation toward AC than classical GTPase-deficient mutants [126]. Several recent orphan GPCR structures (e.g., GPR3 and GPR55) further demonstrate that GPCRs can be directly activated by endogenous lipids [127,128].

Other studies have shown that the cellular abundance of G_i_ has functional consequences. For example, in end-stage human heart failure, upregulation of Gα_i2_ is correlated with markedly reduced AC activity [129], a phenomenon consistent with the enhanced PTx effect observed when Gα_i_ is overexpressed.

## 5. Gα_i_-Dependent but cAMP-Independent Signaling Pathways

PTx not only disrupts the G_i_-mediated inhibition of AC but also interferes with a broad array of G_i_-dependent yet cAMP-independent signaling cascades. The central event is that PTx, through ADP-ribosylation of Gα_i_, prevents dissociation of the heterotrimeric G protein complex, thereby blocking the release of Gβγ subunits. Consequently, many signaling pathways initiated by free Gβγ are globally silenced.

**Figure 7:**
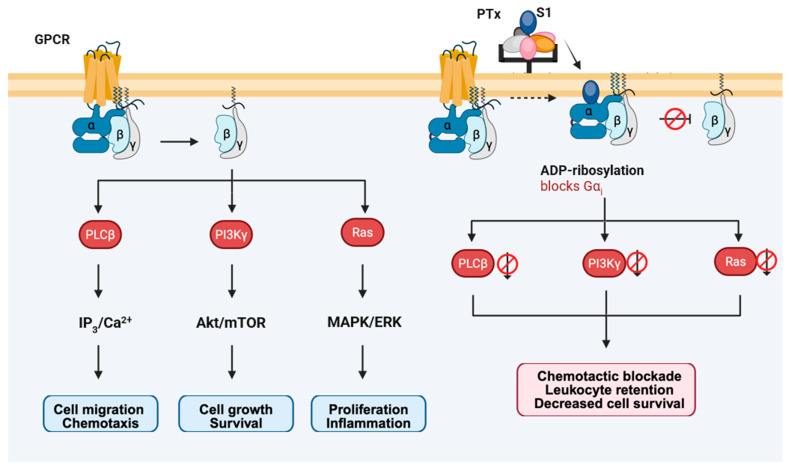
PTx-mediated blockade of G_i_-dependent, cAMP-independent signaling pathways. Under normal conditions, activation of G_i/o_-coupled GPCRs leads to dissociation of the heterotrimer and release of Gβγ subunits, which initiate three major signaling modules: PLCβ–IP_3_/Ca^2+^–Rap1 for chemotaxis, PI3Kγ–Akt/mTOR for growth and survival, and Ras–MAPK/ERK for proliferation and inflammation. PTx ADP-ribosylates Gα_i_ and prevents heterotrimer dissociation, thereby blocking Gβγ release and globally silencing these noncanonical G_i_ pathways. As a result, cells exhibit impaired chemotaxis, leukocyte retention, diminished survival signaling, and defective proliferative responses.

These noncanonical G_i_ pathways are essential for cell growth, migration, and survival, and their loss explains multiple pathological manifestations observed after PTx exposure. The following sections describe how this inhibition impacts key pathways—particularly the mitogen-activated protein kinase/extracellular signal-regulated kinase (MAPK/ERK) pathway, the phosphoinositide 3-kinase–protein kinase B–mechanistic target of rapamycin (PI3K–Akt–mTOR) axis, and the phospholipase Cβ–Ca^2+^–Ras-related protein 1–talin/kindlin (PLCβ–Ca^2+^–Rap1–talin/kindlin) signaling axis (Figure 7), and how these disruptions are manifested at the physiological level as impaired chemotaxis, leukocytosis, and histamine sensitization.

### 5.1. Disruption of MAPK/ERK Signaling

PTx provides a classic example of a G_i_-dependent yet cAMP-independent modulation of MAPK/ERK signaling. In normal GPCR signaling, the Gβγ subunits released from activated G_i/o_ proteins act as the proximal triggers for Ras or PI3K activation, thereby initiating ERK1/2 phosphorylation and downstream proliferative responses [130,131]. This Gi–Gβγ–MAPK coupling has been directly demonstrated for Gi-coupled receptors such as the melatonin MT_1_R and MT_2_R, where ERK and c-Jun N-terminal kinase (JNK) activation proceeds via a PTx–sensitive, Gβγ-, Ras/Rac-, and Src-dependent mechanism [132]. This inhibition is particularly evident in GPCR-mediated responses. Agonists targeting α_2_-adrenergic receptors (α_2_AR) [133], opioid receptors [134], or chemokine receptors [135] normally activate ERK through Gβγ–Ras or Gβγ–PI3K signaling modules. Pretreatment with PTx abolishes this ERK activation. In contrast, receptors that signal through G_s_ remain capable of activating ERK even in the presence of PTx [136].

In cardiac fibroblasts, angiotensin II couples the AT1 receptor to G_i_ proteins, and the released Gβγ subunits sequentially activate Src family tyrosine kinases, the Shc–Grb2–Sos complex, and the Ras/Raf-1 module, thereby driving rapid ERK phosphorylation and promoting DNA synthesis [137]. The insulin-like growth factor-I receptor (IGF-IR) can directly recruit and activate G_i_-family heterotrimeric G proteins, leading to the dissociation of Gα from Gβγ; the liberated Gβγ, together with subsequently recruited β-arrestin, functions as a scaffold to facilitate ERK/MAPK activation [138].

### 5.2. Impact on the PI3K–Akt–mTOR Pathway

The PI3K–Akt–mTOR axis, which is tightly integrated with MAPK signaling, is likewise regulated by G_i_–Gβγ inputs. Under normal conditions, activation of GPCRs leads to the release of Gβγ subunits that recruit and activate PI3Kγ [139,140], driving Akt phosphorylation and downstream mTOR signaling to support cellular metabolism, survival, and growth [139,141]. PTx blocks this process by ADP-ribosylating Gα_i_ and preventing Gβγ release, thereby uncoupling these receptors from the PI3K–Akt–mTOR pathway and markedly attenuating intracellular growth and survival signals [142].

This dependence is well documented in neuronal models (e.g., PC12 cells and cortical neurons) in the nervous system. Nerve growth factor (NGF)-driven relief of Tuberous Sclerosis Complex 2 (TSC2) inhibition (via PI3K/Akt) includes a PTx-sensitive G_i/o_/Gβγ component, thus positioning G_i/o_ upstream of the TSC2 gate that controls mTOR signaling output [143]. In PC12 cells, stimulation of the muscarinic M_4_ receptor (M_4_R) induces mTOR/p70S6K phosphorylation through a G_i/o_-dependent pathway, an effect that is almost completely abolished by PTx treatment [144]. Similarly, PTx attenuates NGF-induced tuberin (TSC2) phosphorylation (most evident at early time points), thereby suppressing mTOR activation; Gβγ scavenging/interference (e.g., transducin) or RGS overexpression produces comparable outcomes. In contrast, epidermal growth factor (EGF) can activate PI3K/Akt independently of G_i_, maintaining TSC2 phosphorylation even in the presence of PTx, further underscoring the selective reliance of the NGF–mTOR link on functional G_i_ signaling. Beyond neuronal NGF signaling, G_i_-coupled receptors such as α_2_-adrenergic and muscarinic receptors can activate the PI3K–Akt pathway via Gβγ release [143].

### 5.3. Disruption of the PLCβ–Ca^2+^–Rap1–Talin/Kindlin Pathway

Beyond regulating the MAPK/ERK and PI3K–Akt–mTOR axes, G_i_–Gβγ signaling also controls cell polarization, adhesion, and migration through activation of the PLCβ–Ca^2+^–Rap1–talin/kindlin module [145].

Under physiological conditions, activation of G_i_-coupled chemokine receptors such as CXCR1/2 and FPR leads to the release of Gβγ subunits, which stimulate PLCβ2/3 to generate IP_3_, thereby inducing intracellular Ca^2+^ mobilization and activating PKC [145,146]. Ca^2+^ and diacylglycerol (DAG) generated downstream of chemokine receptor–PLC signaling primarily activate calcium and diacylglycerol-regulated guanine nucleotide exchange factor I (CalDAG-GEFI), a Rap1 guanine nucleotide exchange factor, thereby inducing Rap1 activation. Active Rap1 then interacts with Rap1–GTP–interacting adaptor molecule (RIAM), which promotes talin recruitment to CD18. In parallel, kindlin-3 also binds to CD18; together, talin and kindlin-3 induce the high-affinity conformation of β2 integrins, enabling strong ligand binding and supporting leukocyte adhesion and migration [147].

## 6. Gα_i_-Independent Activities of PTx: B Oligomer-Mediated Signaling and Immune Modulation

The pathogenic activity of PTx extends far beyond the ADP-ribosyl transferase activity of its S1 subunit. In addition to disrupting both cAMP-dependent and cAMP-independent G_i_ pathways, the PTx B-oligomer possesses substantial receptor-binding and signal-modulating functions on its own. As a multivalent ligand, the B-oligomer can crosslink multiple receptors on the host cell surface and initiate complex immune signaling even in the absence of catalytic activity [47].

**Figure 8:**
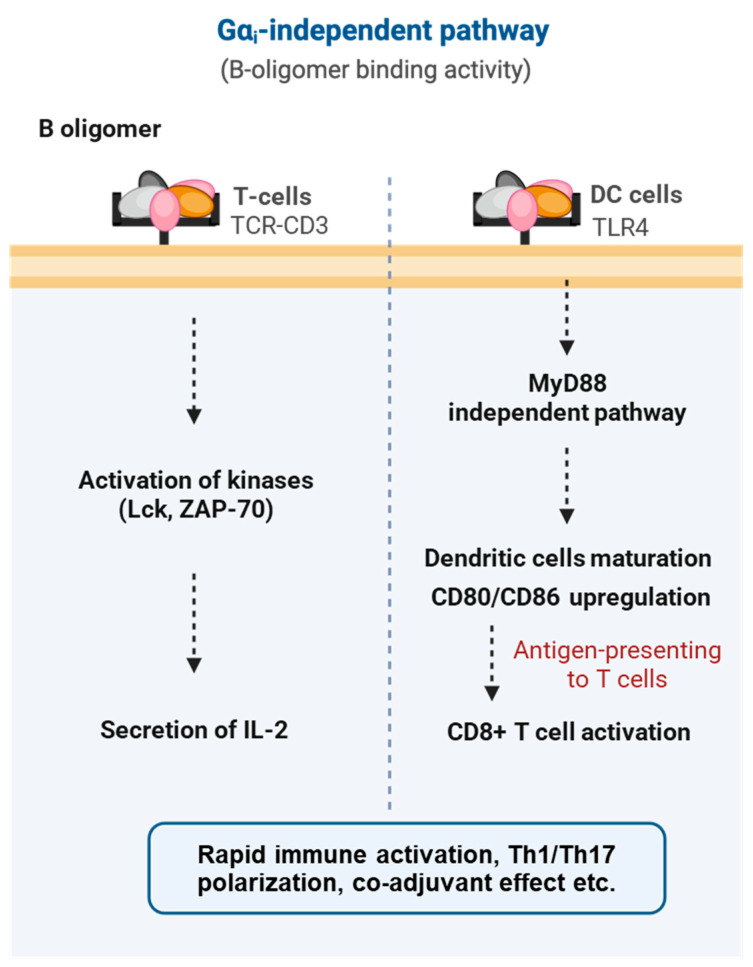
Gα_i_-independent, B oligomer-mediated signaling pathways of pertussis toxin. The B-oligomer of pertussis toxin engages multiple immune and structural cell types independently of Gα_i_ ADP-ribosylation. In T cells, B-oligomer binding to the TCR–CD3 complex triggers rapid kinase activation (Lck, ZAP-70) and promotes IL-2 secretion. In dendritic cells, interaction with TLR4 activates a MyD88-independent maturation pathway, leading to CD80/CD86 upregulation, enhanced antigen presentation, and subsequent CD8^+^ T-cell activation.

Unlike the enzymatic inactivation mediated by the S1 subunit, the PTx B-oligomer can directly bind to host cell surface receptors and initiate intracellular signaling. As a multivalent ligand, the B-oligomer is capable of crosslinking immune receptors and triggering ligand-independent activation [148]. One of the earliest activities identified was its direct action on the T-cell receptor (TCR) complex. By crosslinking TCRs, the B-oligomer activates the downstream tyrosine kinases Lck and ZAP-70, induces Ca^2+^ influx, and stimulates transcription factors such as NF-κB and NF-AT, ultimately driving robust secretion of cytokines including IL-2. Consequently, PTx has long been recognized as a potent T-cell mitogen [148,149] (Figure 8).

Beyond T cells, the B-oligomer also interacts with Toll-like receptor 4 (TLR4) on dendritic cells and macrophages, inducing their maturation through a MyD88-independent pathway [150,151]. This process upregulates costimulatory molecules (CD80/CD86) and promotes the production of IL-6 and IL-12, thereby driving a Th1-skewed immune response [151,152]. Overall, these findings demonstrate that the B-oligomer can directly activate both innate and adaptive immunity without requiring ADP-ribosylation, enhancing antigen presentation and promoting polyclonal T-cell expansion [151,153].

## 7. Conclusions and Future Perspectives

The pathogenic effects of PTx stem from its canonical and noncanonical mechanisms. The S1 subunit of PTx catalyzes the ADP-ribosylation of Gα_i/o_, irreversibly uncoupling them from GPCRs. This modification locks Gα_i_ in an inactive, GDP-bound state, preventing the G_i/o_ family from inhibiting AC activity and leading to elevated intracellular cAMP levels. Excessive cAMP activates PKA and EPAC, disrupting cellular functions and suppressing immune activity, contributing to symptoms like leukocytosis and metabolic dysregulation in pertussis.

PTx also affects cAMP-independent signaling by locking Gα_i_ in the “off” state, which restricts the release of Gβγ subunits that normally mediate various signaling events, including PI3K activation and MAPK cascades. This silencing of Gβγ-dependent processes explains PTx’s inhibition of chemokine-driven leukocyte responses and impairs neutrophil functions, hindering bacterial clearance. Additionally, the B oligomer of PTx serves as an immunomodulatory agent, acting as a T-cell mitogen and engaging TLR4 on dendritic cells and macrophages, contributing to the immune regulatory landscape during infection. PTx’s dual effects—pro-inflammatory via TLR4 activation and immunosuppressive through cAMP elevation—illustrate its complex role in immune modulation.

Constitutive G_i_ signaling is another key target of PTx. By inactivating G_i/o_ proteins, PTx removes the tonic inhibitory signaling from G_i_-coupled receptors, altering functional states in immune and endocrine cells. This contributes to dysregulation in pertussis, highlighting the active role of G_i_ proteins in maintaining homeostasis rather than being mere switches.

PTx’s virulence is enhanced in conjunction with ACT, as both toxins elevate cAMP but act through different mechanisms, leading to a coordinated suppression of bacterial clearance and dysregulated inflammation. This synergy highlights the complex interplay between PTx and ACT in modulating host immune responses and bacterial clearance.

Overall, PTx serves as a model for understanding AB-type bacterial toxins and their effects on host signaling pathways. Continued research on PTx will advance efforts against pertussis and improve our understanding of G protein signaling and immune regulation in health and disease.

## 8. Materials and Methods

### 8.1. Structural Visualization and Molecular Docking

The structural visualizations in Figure 2 were generated using PyMOL 3.1.4 (Schrödinger, LLC, New York, NY, USA) software. The structures of the full PTx and the S1 subunit in its ATP-activated state (Figure 2A) were visualized using the PDB entry 1PRT and 7SKY, respectively. For the visualization of the NAD^+^ binding site (Figure 2B), key residues were highlighted using the 7SKY PDB file entry. The structural complex model shown in Figure 2C–E was generated using the AlphaFold web server [76] (accessible at https://alphafoldserver.com; accessed on 1 August 2025). The amino acid sequences for the PTx S1 subunit, Gα_i1_, Gβ_1_, and Gγ_2_, and NAD and GDP molecules were provided as inputs for the complex simulation. From the five top-ranked models generated by the server, the one that showed the highest consistency with reported S1-Gα_i_ interactions [58,75] was manually selected for subsequent visualization and analysis.

### 8.2. Cell Culture and Transfection

Human embryonic kidney 293 cells (HEK293; ATCC CRL-1573, American Type Culture Collection, Rockville, MD, USA) were maintained under standard conditions. For cAMP assays, cells were seeded into 6-well plates at a density of 3.5 × 10^5^ cells/mL in 2 mL medium per well, yielding 7 × 10^5^ cells/well. On the same day, transient transfection was performed using polyethyleneimine Star™ (PEI; Tocris Bioscience, Bristol, UK). Each well received 400 ng of the cAMP-Glosensor-22F plasmid (Promega, Madison, WI, USA), 400 ng of a single GPCR-encoding plasmid (either β_2_-AR, D_1_R, D_2_R, MT_1_R, or MT_2_R; UMR cDNA Resource Center, Rolla, MO, USA), and 400 ng of either Gα_i1_ or empty vector control (pcDNA3.1).

### 8.3. cAMP Bioluminescence Assay

Twenty-four hours post transfection, cells were trypsinized and seeded into white-bottom 96-well plates at a density of 4 × 10^4^ cells/well. Pertussis toxin (PTx; List Biological Laboratories, Campbell, CA, USA) was added at a final concentration of 100 ng/mL, and cells were incubated for an additional 24 h. Prior to assay initiation, growth medium was replaced with a labeling medium composed of HBSS buffer supplemented with 20 mM HEPES (pH 7.5) and 2% (*v*/*v*) GloSensor™ cAMP Reagent stock solution. Plates were incubated for 30 min at 37 °C, followed by 15–20 min of equilibration at room temperature. Bioluminescence was measured using a SpectraMax microplate reader (Molecular Devices, San Jose, CA, USA) with an emission wavelength of 570 nm.

### 8.4. Statistical Analysis

Data shown in the figures are the mean  ±  SD of three independent experiments performed in triplicate. The data sets were analyzed by using multiple unpaired *t*-tests, two-tailed, with 95% confidence (ns indicates not significant; *, *p* ≤ 0.05).

### 8.5. Materials

All experiments were performed using Human embryonic kidney 293 cells (HEK293, ATCC CRL-1573) obtained from the American Type Culture Collection (Rockville, MD, USA). Polyethyleneimine StarTM (PEI) was purchased from Tocris, while plasmids were purchased from UMR cDNA Resource Center (Rolla, MO, USA). Pertussis toxin (PTx) was purchased from List Biological Laboratories (Campbell, CA, USA), and reagents for cell culture were purchased from Thermofisher Scientific (Waltham, MA, USA).

### 8.6. Use of Generative AI

Generative AI was used to assist in the editing and formatting of this manuscript. No AI tools were used in data analysis or interpretation.

## Figures and Tables

**Table 1:** Major toxins of *B. pertussis*.

Toxin	Pertussis Toxin (PTx)	Adenylate Cyclase Toxin (ACT)	Tracheal Cytotoxin (TCT)	Dermonecrotic Toxin (DNT)	Lipopolysaccharide (LOS)
Molecular Structure	•105 kDa heterohexamer; •S1: (26 kDa) •S2–S5: (22, 22, 12, 10 kDa) •Ratio: 1:1:1:2:1	•Bifunctional protein; •AC domain: adenylate cyclase-like •RTX domain: pore-forming (Ca^2+^-dependent)	•Peptidoglycan fragment •~920 Da	•Single-chain protein •~140 kDa •Heat-labile •Deamidase activity	•Lipooligosaccharide •Lipid A + core oligo •Lacks O-antigen repeats •Outer membrane component
Primary Target/Receptor	•Glycoprotein receptors •A conserved Cys in C-terminal region of Gα_i_/Gα_o_/Gα_t_ proteins	•CD11b/CD18 integrin (CR3 complement receptor) •Phagocytes (preferential) •Host calmodulin	•Ciliated respiratory epithelium •Tracheal cells •Bronchial epithelium •NOD1 receptor pathway	•Rho family GTPases (RhoA, Rac, Cdc42) •CaV3.1 calcium channels •Neurotropic effects	•TLR4/MD-2 complex •CD14 co-receptor •Innate immune cells
Mechanism of Action	•ADP-ribosyltransferase activity, NAD^+^ → ADP-ribose transfer to the Cys residue •Uncouples G_i_/G_o_-receptor signaling	•Calmodulin-dependent AC activation •ATP → cAMP conversion •Membrane pore formation, K^+^ efflux, Ca^2+^ influx	•Nitric oxide (NO) production •Apoptosis induction •Ciliary dysfunction	•Deamidation of Gln → Glu •Constitutive Rho activation •Disrupts GTPase cycle •Calcium channel binding	•TLR4 activation •MyD88/TRIF signaling •NF-κB activation •Pro-inflammatory cascade
Physiological Effects ^a^	•↑ Intracellular cAMP •Lymphocytosis •Insulin hypersecretion •Histamine sensitization •Vascular permeability	•↑ cAMP in phagocytes •Impaired chemotaxis •Reduced phagocytosis •Apoptosis (high doses) •Hemolysis	•Ciliary stasis •Mucus clearance impairment •Paroxysmal cough •Airway inflammation	•Cytoskeletal reorganization •Stress fiber formation •Focal adhesion assembly •Necrotic skin lesions •Neurological complications	•Fever induction •Inflammatory cytokines ↑ (IL-1β, TNF-α, IL-6) •Acute phase response •Complement activation
Systems Affected ^b^	•Immune •Endocrine •Cardiovascular •Metabolic	•Immune	•Respiratory	•Nervous •Cytoskeletal	•Immune
Refs	[3,4,5,6,8,27,28,29,30,31,32,33]	[16,17,34,35,36,37]	[22,23,37,38]	[14,24,25,39,40]	[26,41,42,43,44,45]

Abbreviations: AC, cAMP, CR3, DAP, GlcNAc; MurNAc, NOD1, RTX, TLR4, MyD88, TRIF, MD-2, TNF-α. ^a^ Physiological effects represent primary documented responses. ^b^ Systems affected indicate major target organ systems based on current literature. Upward arrow (↑) denotes an elevation in levels or activity (e.g., ↑ cAMP indicates increased cAMP levels).

**Table 2:** Review of key functional residues/regions in S1 of PTx.

	Residue/Region	Role in Catalysis	Effect of Mutations on ADP-Ribosyltransferase Activity	References
Catalytic core residues	E129	Essential catalytic glutamic acid; positions ribosyl ring of NAD^+^ at transition state	E129D/G/Q: reduces activity	[63,64,65,66]
	Y8	Universally conserved in several AB toxins (e.g., CTX, ETA, DT)	Not tested, conservation inferred from sequence and structural alignment	[28,60,61,62]
	H35	Enhances nucleophilicity of target cysteine in Gα_i_	Also reduces NAD^+^ glycohydrolase activity	[67,68]
	Q127	Part of conserved Q/EXE motif; supports active site geometry	Reduces activity to ~50%	[58,69]
	S52	Maintains active site conformation (STS motif)	S52F/G/A reduces NAD^+^ glycohydrolase activity	[67]
NAD^+^ binding and positioning	R9	Electrostatic interaction with NAD^+^ diphosphate backbone	R9K/E129G: become detoxified pertussis toxin	[63,70,71]
	W26	Contacts adenylyl ribose; stabilizes NAD^+^-binding loop	Mutation/deletion greatly reduces activity	[72,77]
	S5/S52/T53/Y130	Contact nicotinamide ribose or diphosphate backbone of NAD^+^	Not tested, determined by docking models	[73]
	Y10/H35/L36/Q42/Y63	Other residues that contact β-NAD^+^ (PDB code: 7SKY)	Not tested, determined by crystal structures	[58,74]
Regulatory/Structural control	C41	Forms disulfide bond with C201; keeps enzyme inactive until cytoplasmic reduction	C41S: WT activity; deletion abolishes	[28,78]
	C201	Disulfide bonding with C41	Not tested alone	[28]
	Residues 205–219	Likely link catalytic domain to B-oligomer-binding region	Not tested alone	[75]
	Residues 220–235	Associate with B-oligomer	C219 (S1 amino acids 1–219) showed no association with B-oligomer	[75]
Substrate (Gα_i_)	Y59/Y63	Hydrophobic contacts with Gα_i_ C-terminal tail (IKNNLKECGLF)	Mutation to Ala significantly reduces activity	[58]
	Residues 195–204	Required for high affinity G_t_ trimer protein binding	Activity of C180 (S1 amino acids 1–180) was 2% of S1, while C204 (S1 amino acids 1–204) was 80% of S1.	[75]

Abbreviations: NAD^+^, β-NAD^+^, Gα_i_, G_t_, CTX, ETA, DT, WT, STS motif, Q/EXE motif. Experimental and structural data on key residues for ADP-ribosyltransferase activity, NAD^+^ binding, regulatory control, and substrate recognition are illustrated. Residue numbering corresponds to the S1 subunit sequence (ptxS1 from Uniprot), and mutations are designated using standard single-letter amino acid code. Activity percentages are reported relative to wild-type S1 unless otherwise specified. Crystal structure data are referenced from PDB code 7SKY. Truncated constructs C180, C204, and C219 contain S1 amino acids 1–180, 1–204, and 1–219, respectively.

## Data Availability

No new data were created or analyzed in this study.

## Abbreviations

The following abbreviations are used in this manuscript:

AB	AB-type bacterial toxin
AC	Adenylyl cyclase
ACT	Adenylate cyclase toxin
ADP	Adenosine diphosphate
AoE	Areas of Excellence Scheme
ATP	Adenosine triphosphate
AT1R	Angiotensin II type 1 receptor
β2-AR	Beta-2 adrenergic receptor
β-NAD^+^	Beta-nicotinamide adenine dinucleotide
BvgAS	Bordetella virulence gene regulatory system
Ca^2+^	Calcium ion
CalDAG-GEFI	Calcium and diacylglycerol-regulated guanine nucleotide exchange factor I
cAMP	Cyclic adenosine monophosphate
CCR1	C-C chemokine receptor 1
CR3	Complement receptor 3
CTX	Cholera toxin
DAG	Diacylglycerol
D1R	Dopamine D1 receptor
D2R	Dopamine D2 receptor
DAP	Diaminopimelic acid
DNT	Dermonecrotic toxin
DT	Diphtheria toxin
EGF	Epidermal growth factor
EPAC	Exchange protein directly activated by cAMP
ER	Endoplasmic reticulum
ERK	Extracellular signal-regulated kinase
ETA	Exotoxin A
FPR	Formyl peptide receptor
GlcNAc	N-acetylglucosamine
Gα	G protein alpha subunit
Gα_i_	Alpha subunit of inhibitory G protein
Gβγ	G protein beta-gamma complex
GEF	Guanine nucleotide exchange factor
G_i_	Inhibitory G protein
G_i/o_	Inhibitory G protein family (Gi and Go)
GPCR	G protein-coupled receptor
GPR	G protein-coupled receptor
G_s_	Stimulatory G protein
G_t_	G protein transducin
HBSS	Hank’s balanced salt solution
HEK293	Human embryonic kidney 293 cells
HEPES	4-(2-hydroxyethyl)-1-piperazineethanesulfonic acid
HSF	Histamine-sensitizing factor
IAP	Islet-activating protein
IGF-IR	Insulin-like growth factor I receptor
IL	Interleukin
IP3	Inositol 1,4,5-trisphosphate
JNK	c-Jun N-terminal kinase
kDa	Kilodalton
LOS	Lipooligosaccharide
LPF	Lymphocytosis-promoting factor
MAPK	Mitogen-activated protein kinase
MD-2	Myeloid differentiation factor 2
mTOR	Mechanistic target of rapamycin
MT1R	Melatonin receptor 1
MT2R	Melatonin receptor 2
MurNAc	N-acetylmuramic acid
MyD88	Myeloid differentiation primary response protein 88
NAD^+^	Nicotinamide adenine dinucleotide
NF-AT	Nuclear factor of activated T cells
NF-κB	Nuclear factor kappa-light-chain-enhancer of activated B cells
NGF	Nerve growth factor
NOD1	Nucleotide-binding oligomerization domain 1
PBS	Phosphate-buffered saline
PC12	Pheochromocytoma 12 cells
PEI	Polyethyleneimine
PI3K	Phosphoinositide 3-kinase
PKA	Protein kinase A
PKC	Protein kinase C
PLCβ	Phospholipase C beta
PTx	Pertussis toxin
Q/EXE motif	Glutamine-X-glutamic acid conserved sequence pattern where X represents any amino acid
Rap1	Ras-related protein 1
RIAM	Rap1–GTP–interacting adaptor molecule
RLU	Relative luminescence unit
RTX	Repeats-in-toxin
SD	Standard deviation
STS motif	Serine-threonine-serine sequence motif
S1	Catalytic subunit of pertussis toxin
S1P	Sphingosine-1-phosphate
Src	Proto-oncogene tyrosine-protein kinase Src
TCR	T-cell receptor
TCT	Tracheal cytotoxin
Th1	T helper type 1
Th17	T helper type 17
TLR	Toll-like receptor
TLR4	Toll-like receptor 4
TNF-α	Tumor necrosis factor alpha
TRIF	TIR domain-containing adaptor inducing interferon-β
TSC2	Tuberous sclerosis complex 2
VAAS	Vasoactive amine sensitization
WT	Wild type
